# The guideline implementability research and application network (GIRAnet): an international collaborative to support knowledge exchange: study protocol

**DOI:** 10.1186/1748-5908-7-26

**Published:** 2012-04-02

**Authors:** Anna R Gagliardi, Melissa C Brouwers, Onil K Bhattacharyya

**Affiliations:** 1 University Health Network, Toronto, ON, Canada; 2 McMaster University, Juravinski Hospital Site, Toronto, ON, Canada; 3 St. Michael's Hospital, Toronto, ON, Canada

**Keywords:** Guidelines, Guideline development, Guideline implementation, Research networks, Knowledge exchange

## Abstract

**Background:**

Modifying the format and content of guidelines may facilitate their use and lead to improved quality of care. We reviewed the medical literature to identify features desired by different users and associated with guideline use to develop a framework of implementability and found that most guidelines do not contain these elements. Further research is needed to develop and evaluate implementability tools.

**Methods:**

We are launching the Guideline Implementability Research and Application Network (GIRAnet) to enable the development and testing of implementability tools in three domains: Resource Implications, Implementation, and Evaluation. Partners include the Guidelines International Network (G-I-N) and its member guideline developers, implementers, and researchers. In phase one, international guidelines will be examined to identify and describe exemplar tools. Indication-specific and generic tools will populate a searchable repository. In phase two, qualitative analysis of cognitive interviews will be used to understand how developers can best integrate implementability tools in guidelines and how health professionals use them for interpreting and applying guidelines. In phase three, a small-scale pilot test will assess the impact of implementability tools based on quantitative analysis of chart-based behavioural outcomes and qualitative analysis of interviews with participants. The findings will be used to plan a more comprehensive future evaluation of implementability tools.

**Discussion:**

Infrastructure funding to establish GIRAnet will be leveraged with the in-kind contributions of collaborating national and international guideline developers to advance our knowledge of implementation practice and science. Needs assessment and evaluation of GIRAnet will provide a greater understanding of how to develop and sustain such knowledge-exchange networks. Ultimately, by facilitating use of guidelines, this research may lead to improved delivery and outcomes of patient care.

## Background

### Guideline implementability

Guidelines are syntheses of best available evidence that, along with professional judgment and patient preferences, support decision making by clinicians, managers, and policy makers about the organisation and delivery of healthcare. However, they continue to be underused [1-7]. Research has shown that guideline format and content influence perceptions about and use of guidelines. Specifically, these intrinsic guideline qualities have been shown to promote greater understanding of how users are to apply the recommendations, stimulating confidence in users' ability to practice the recommended behavior, leading to greater intent to use guidelines and actual use [8-13]. Thus, use of guidelines might be optimised by improving their format and content. The concept of implementability was first defined by Shiffman as characteristics of guidelines that may enhance their implementation by users, and he issued consensus recommendations for generating guidelines with actionable wording [14]. To further investigate the concept of implementability, we reviewed the medical literature to identify features desired by different users or associated with guideline use [15]. The guideline implementability framework included 22 elements organised within eight domains: adaptability, usability, relevance, validity, applicability, communicability, resource implications, implementation, and evaluation (Table 1). Our analysis of guidelines on various clinical indications judged by experts as high quality found that most did not contain implementability elements, highlighting numerous opportunities to potentially improve guideline development and use by integrating one or more of these elements.

**Table 1:** Guideline implementability framework

Domain	Definition	Element	Examples
Adaptability	The guideline is available in a variety of versions for different users or purposes	Sources	Internet, peer-reviewed journal
		
		Versions	Full text, summary, print, digital
		
		Users	Tailored for patients or caregivers

Usability	Content is organised to enhance the ease with which the guideline can be used	Navigation	Table of contents
		
		Evidence	Narrative, tabulated, or both
		
		Recommendations	Narrative, graphic (algorithms), or both; recommendation summary (single list in full or summary version rather than dispersed)

Validity	Evidence is summarised and presented such that its quantity and quality are apparent	Number of references	Total number of distinct references to evidence upon which recommendations are based
		
		Evidence graded	A system is used to categorise quality of evidence supporting each recommendation
		
		Number of recommendations	Total number of distinct recommendations

Applicability	Information is provided to help interpret and apply guidelines for individual patients	Clinical considerations	Information such as indications, criteria, risk factors, and drug dosing that facilitates application of the recommendations explicitly highlighted as tips or practical issues using subtitles or text boxes or summarised in tables and referred to in recommendations or narrative

Communicability	Resources for providers or patients to inform, educate, support, and involve patients	Inform, educate, support	Informational, educational, or supportive resources for patients/caregivers, or contact information (phone, fax, email, or URL) for such resources
		
		Decision making	Questions or tools for clinicians to facilitate discussion with patients, or decision aids to support patient involvement

Relevance	The focus or purpose of the guideline is explicitly stated	Objective	Explicitly stated purpose of guideline (clinical, education, policy, quality improvement)
		
		Stakeholders	Specify who would deliver (individuals, teams, departments, institutions, managers, policy makers, internal/external agents) and receive the services (specify type of patients)
		
		Needs	Identification of stakeholder needs, perspectives, interests, or values

Resource implications	Anticipated changes, resources, and competencies required to adapt and accommodate guideline utilisation are identified	Technical	Equipment or technology needed, or the way services should be organised
		
		Regulatory	Industrial standards for equipment or technology, or policy regarding their use
		
		Human resources	Type and number of health professionals needed to deliver recommended services
		
		Professional	Education, training, or competencies needed by clinicians/staff to deliver recommendations
		
		Workflow	Anticipated changes in workflow or processes during/after adoption of recommendations
		
		Costs	Direct or productivity costs incurred by acquiring resources or training to accommodate guidelines, or as a result of service reductions during transition from old to new processes

Implementation	Processes for planning and applying local strategies to promote guideline utilisation are described	Identify barriers	Individual, organisational, or system barriers that could challenge adoption, or instructions for local needs assessment of guideline users
		
		Tailor guideline	Instructions, tools, or templates to tailor guideline/recommendations for local context
		
		Integrated tools	Point-of-care templates/forms (clinical assessment, standard orders) to integrate guidelines within care delivery processes
		
		Promote utilisation	Possible mechanisms by which to promote guideline utilisation

Evaluation	Processes for evaluating guideline implementation and utilisation are described	Implementation	Methods for evaluating the implementation process
		
		Utilisation	Audit tools or performance measures/quality indicators to assess the organisation, delivery, and outcomes of guideline-recommended care

### Collaborating to investigate guideline implementability

Further research is needed to operationalise the implementability concept by developing and evaluating implementability tools. Validation and definitive testing of tools and interventions based on the implementability framework will require considerable collaboration with guideline developers, implementers, and users to more strategically generate knowledge, build a consolidated research base, and accelerate its application into practice. To accomplish this, a more formalised network is needed to leverage and sustain existing relationships and resources and to create capacity for collaboration on research and application of guideline implementability. With funding from the Canadian Institutes of Health Research, we are launching the Guideline Implementability Research and Application Network (GIRAnet). Partners include guideline agencies in Canada, United States, Australia, New Zealand, The Netherlands, Italy, Scotland, England, and Finland and the Guidelines International Network (G-I-N). G-I-N is a nonprofit association of guideline developers, implementers, and users, including 94 organisational and 76 individual members from 46 countries http://www.g-i-n.net. G-I-N represents a natural gateway for research collaboration with stakeholders. The purpose of the network is to

1. create a formal and identifiable collaborative of those interested in guideline implementability;

2. generate a user-informed research agenda based on guideline implementability;

3. leverage available capacity to develop, implement, and evaluate tools and interventions based on the implementability framework;

4. plan for and establish a sustainable environment that will enable ongoing evaluation of implementability tools and interventions;

5. accelerate the translation of this new knowledge into guideline development and quality-improvement practices.

### Network design

#### Approach

A review of research in various disciplines relevant to collaborative partnerships (including management networks; interprofessional health-services research, teamwork, and collaboration; continuity of care; knowledge translation; communities of practice; and quality-improvement collaboratives) found that collaboration can only be effective with investment in infrastructure that, at a minimum, includes a dedicated individual who will communicate with and engage participants, facilitate the creation of linkages, organise forums for interaction that include in-person meetings, and actively support the development of strategic plans and the undertaking of network activities from which participants derive benefit [16-32]. We designed the structures and activities of GIRAnet to align with this evidence.

### Infrastructure and activities

The GIRAnet structure includes the administrative site, steering committee, research group, and various levels of membership. The *administrative site *will lead and coordinate all network activities; lead the development of implementability tools; and support distributed development, implementation, and evaluation of implementability tools. Prioritisation and direction for network activities will be provided by a *steering committee *that will advise on these matters. It is comprised of representatives from guideline-development agencies in nine partner countries plus G-I-N. Some examples of network activities include compiling a directory of existing implementability tools; developing and sharing guidance on the development and use of implementability tools, including toolkits and training sessions; conducting a needs assessment of guideline developers and implementers to better understand the resources needed to develop implementability tools; planning mechanisms by which to sustain a research network focused on guideline implementation; and generating a user-informed research agenda. A *research group *will provide input on research activities, including methods for the development and evaluation of tools and interpretation of evaluation findings. It is comprised of the manuscript co-authors, who are collaborating on operationalising the implementability concept by focusing their efforts on differing domains, and others who undertake academic research related to the development, implementation, and use of actionable guidelines. *Members *refers to anyone interested in guideline development, implementation, or related research who may wish to remain informed about network activities and products or take a more active role and participate in the evaluation of implementability tools as described in the next section.

## Research design

### Approach

The specific mandate of GIRAnet is to enable research that will develop, implement, and evaluate tools and interventions based on the implementability framework. The overall research plan is based on the MRC Framework for Developing and Evaluating Complex Interventions [33]. This approach includes five steps: development, pilot testing, evaluation, reporting, and implementation. However, evaluation must be informed by, and tailored to, the results of development and pilot testing; therefore, the proposed work will take place over three years and address development and pilot testing and planning for subsequent evaluation (Table 2). We will focus on identifying and/or developing implementability tools for guidelines on arthritis, cancer (breast, prostate, colorectal, lung), chronic obstructive pulmonary disease, depression, diabetes, ischemic heart disease, and stroke. These are relevant to national funding priorities in Canada, affect both men and women, and are the major cause of death and disability worldwide.

**Table 2:** Overall methodological approach based on the MRC Framework for the Development of Complex Interventions

Steps in MRC Framework	This proposal			Future research
		
	Year 1	Year 2	Year 3	
Development	Create implementability tool prototypes based on guideline exemplars and medical literature	---	---	---

Pilot testing	---	Refine prototypes by testing with/to learn: • Developers, data collection/inclusion • Users/tool impact	---	---

Evaluation	---	---	Conduct small- scale study to plan for large-scale evaluation	Conduct large-scale multisite study to evaluate impact of guidelines featuring implementability tools

Reporting	---	---	---	Disseminate findings to guideline developers and researchers through publications, meetings

Implementation	---	---	---	Create and implement kits for guideline developers to include implementability tools in guidelines

### Implementability domains

We will focus on three specific implementability domains/elements not under investigation by others, for which there is little or no research and, according to our research, are seldom included in guidelines (Table 3). *Resource Implications *refers to equipment or technology needed; industrial standards; policies governing their use; type and number of health professionals needed to deliver services; education, training, or competencies needed by staff to deliver services; anticipated changes in workflow or processes during or after adoption; and costs. Health professionals we interviewed said that this type of information would help them prepare for the impact on practice of adopting new guidelines [34]. *Implementation *refers to identifying barriers associated with adoption and selecting and tailoring implementation strategies that address those barriers. Our research found that professionals who fund, manage, and deliver health services lack knowledge about how to implement guidelines. Interviews with policy makers, managers, health professionals, and researchers revealed confusion about responsibility and approaches for implementation [32]. *Evaluation *refers to tools based on performance measures to assess baseline and postintervention compliance with guidelines. Our research found that self-assessment tools based on guidelines are lacking [35]; physicians said they lacked tools to monitor their own performance [36], and providing physicians with self-assessment tools and instructions resulted in identification of learning needs in 66.7% of patient cases they reviewed and modifications in intended care plans for 34.2% of those cases [37].

**Table 3:** Implementability domains and elements of interest

Implementability domain/elements	Definition	Examples of tools	Knowledge to Action phase*
Accommodation • Technical • Regulatory • Human resources • Professional • Workflow process • Costs	Equipment or technology needed; industrial standards; policies governing their use; type and number of health professionals needed to deliver services; education, training, or competencies needed by staff to deliver services; anticipated changes in workflow or processes during or after adoption	• Literature search strategies for identifying these elements for condition-specific guidelines • Template statement for inclusion in guidelines • Strategy for whom to engage in guideline development to enable use of an integrated knowledge-translation strategy and governance structure	Adapting Knowledge to Local Context; Assessing Barriers, Facilitators of Knowledge Use (phases 2, 3)

Implementation • Barriers and facilitators • Strategies • Tailoring	Identifying individual, organisational, and system barriers associated with adoption; selecting and tailoring implementation strategies that address barriers	• Literature search strategies for identifying barriers • Criteria and algorithms for selecting interventions • Options for tailoring interventions • Template statement for inclusion in guidelines • Surveys to facilitate systematic barrier analysis and mitigation	Assessing Barriers, Facilitators of Knowledge Use; Selecting, Tailoring, and Implementing Interventions (phases 3,4)

Evaluation • Performance measures • Benchmarks • Evaluation processes	Tools based on performance measures that can be used by organisations or individuals to assess their baseline and postintervention compliance with recommendations	• Program evaluation kit • Self-audit kit	Monitoring Knowledge Use, Evaluating Outcomes, Sustaining Knowledge Use (phases 5, 6, 7)

*From: Graham ID, Logan J, Harrison MB, Straus SE, Tetroe J, Caswell et al.: **Lost in knowledge translation: time for a map? ***J Cont Ed Health Prof *2006; **26**:13-24.

### Theoretical framework

**Figure 1:**
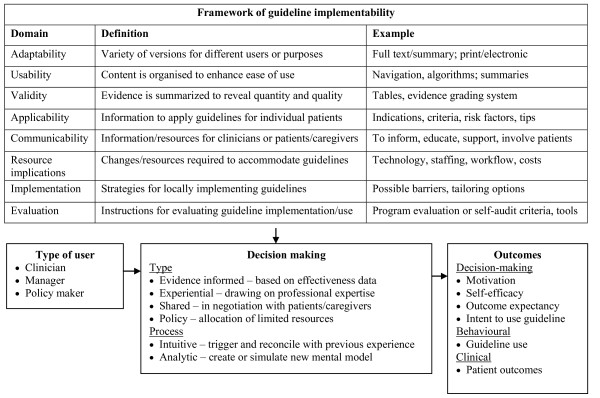
**Theoretical framework**. This framework will guide data collection and analysis, and study findings will confirm and extend its components. The framework proposes that different types of guideline users would apply information reflecting implementability domains in different ways. The way they interpret and use the information may vary by type of decision making, and by decision making process. Use of the implementability information in various ways may lead to different potential outcomes.

Cognitive science theory suggests that guidelines may be difficult to use because they present complex information that prescribes action that may not match clinical circumstances or user preferences, and individual knowledge and experience shape the way that information in a guideline is processed [38]. Guidelines featuring implementability tools may overcome these limitations to facilitate guideline interpretation and use. However, we need greater insight on the mechanism(s) by which this occurs to better understand how guideline format and content can be optimised. Through literature review, we compiled a conceptual framework that describes how implementability elements may influence type and process of decision making and guideline use and outcomes. Implementability elements may support various types of decisions: evidence informed (effectiveness data), experiential (professional expertise), shared (negotiation with patients/caregivers), and policy decision making (resource allocation) [15]. Implementability elements may support different types of decision-making processes: intuitive--trigger or reconcile with previous experience--and analytic--create or simulate new mental model [16-20]. This framework will guide data collection and analysis. Study findings will validate and extend the framework and help us to refine prototype implementability tools (Figure 1).

### Phase one: identify and develop prototype tools

The content of international guidelines on indications of interest will be analysed to identify exemplar implementability tools reflecting domains of interest (Resource Implications, Implementation, Evaluation). Content analysis describes phenomena in written, verbal, or visual communication to generate or validate a framework or model [39]. We will use a directed approach [40]. This means that explicit content in guidelines will be coded using elements from the implementability framework. We used these methods when we first examined guidelines for implementability [15]. To supplement exemplars, or if none are identified, literature searches will be conducted to identify information reflecting implementability elements with which to populate tools and templates. For example, if guidelines for arthritis management contained little or no resource-implications information, we will search the medical literature to identify relevant technical, regulatory, human resource, and workflow issues. Similarly, if no diabetes guidelines contained performance-assessment instructions, we will search the medical literature for examples of assessment measures with which to create program or evaluation tools. The search strategies themselves will ultimately be used in toolkits for developers, along with indication-specific and generic implementability tools that are relevant across disease indications so that they can find similar information for other guidelines.

### Phase two: pilot test and refine prototype tools

Cognitive interviewing will be used to understand how developers can best integrate implementability tools in guidelines and how health professionals can use them for interpreting and applying guidelines. Findings will be used to refine indication-specific prototypes and generic templates in the instructional manual. This approach is based on cognitive theory to understand human information processing (attention span, word recognition, language processing, action, problem solving, reasoning) and has been used widely to understand how respondents interpret survey questions and for usability testing of information technology [41]. It therefore can be applied to study how developers and users perceive the content, format, use, and impact of implementability tools. The fundamental procedure is the semistructured interview. Interviewing can take place concurrently or subsequent to the respondent reviewing the prototype implementability tools in question, and we will use both to minimise recall bias. The process involves probing, where respondents paraphrase information, define meanings, and identify information that is difficult to understand or use. For example, questions may address the content and format of the tool, feasibility of using the tool and anticipated acceptability of the tool by colleagues, and perceptions of how the tool will be used. It also involves think-aloud protocols, where respondents are asked to verbalise their thought processes as they read and interpret and consider information in the tool, its meaning, and how it will be used. The interviewer classifies problems and determines how the tool should be refined, including aspects that should be either removed or modified and how, and the reason for the change. Types of problems include lexical (understanding of the meaning of words and phrases), inclusion/exclusion (scope of the information), temporal (time taken to read, interpret, and consider information), logical (relevance of the information in general, and as presented), and computational (any problems that do not fall in above categories). Qualitative methods will be used for sampling, data collection, and data analysis [42].

### Phase three: pilot test tool evaluation

Through analysis of exemplar guidelines and supplementary literature search, phase-one development will assemble and create indication-specific and generic implementability tools and templates. Through interviews with guideline developers and users, phase-two pilot testing will assess the feasibility of, and resources needed to develop, implementable guidelines featuring those tools and the impact of those implementability tools on attitude, confidence, outcome expectancy, and intent to use guidelines. An international, multisite quantitative and qualitative study can then be conducted to evaluate the impact of newly developed or modified guidelines featuring implementability tools on actual behaviour or use of guidelines. While before-after observational design is not the strongest test of impact, this is methodologically the most appropriate next step in the evaluation of implementability tools [43]. It will enable comparisons of impact across type of implementability tool and clinical indication and inform the planning of a future, more definitive, time series or pragmatic randomised study to evaluate the cost-effectiveness of implementable guidelines versus usual guidelines or other type of intervention. Furthermore, evaluation in multiple international sites enabled through G-I-N and GIRAnet means that fewer participating health professionals are needed from each site, recruitment and data collection can take place in a shorter period of time, and results are more broadly generalisable and more rapidly translated to practice. However, the conduct of such an international multisite study will be logistically challenging. Moreover, conduct will be dependent on the findings of phase-one development and phase-two pilot testing. Therefore, an intermediate step is needed to prepare for phase-three evaluation. This will take the form of a small-scale pilot test of the planned evaluation, including quantitative analysis of chart-based behavioural outcomes and qualitative analysis of interviews with participants to gather feedback that will inform and refine the subsequent full-scale evaluation.

## Discussion

Infrastructure funding to establish GIRAnet will be leveraged with the in-kind contributions of collaborating national and international guideline developers to advance our knowledge of implementation practice and science. While establishing and maintaining such a network will be challenging, collaborating partners have expressed enthusiasm for greater sharing of information about best practices related to guideline development and implementation, which is imperative to its success.

Findings will further implementation practice by translating implementability theory into action. We will do so by developing implementability tools with and for developers in the field and then more broadly disseminating and implementing those tools to guideline developers through GIRAnet, G-I-N, and various other media and forums. Findings could be used to develop checklists or tools by which to inform guideline development and evaluate guidelines. Developers can use this knowledge to refine their programs, practices, and products and understand how implementability content can be collected and integrated, highlighting resource implications they must consider when applying this approach for promoting guideline use. Thus, we will promote application of implementability tools in real-world health-system contexts. This knowledge can also inform guideline development standards and instructional manuals. We recently reviewed six such instructional manuals and found that they included little implementability information (manuscript submitted).

Findings will further implementation science by exploring the views of different users to elucidate how implementability tools would be interpreted and used, leading to a greater understanding of their potential impact and of measures by which impact could be evaluated in future studies, and validating a theoretical framework of implementability. By developing and evaluating an alternative mechanism of implementing guidelines that can be intrinsically introduced at the time of guideline development, we advance our understanding of how to link implementation with development. By investigating and tailoring methods for evaluating prototypes, we may develop methods for rapid-cycle testing that may be widely applicable for assessing other knowledge-based interventions. In particular, we will use the findings to inform ongoing research, leading to definitive testing of implementability tool impact through future time series or randomised studies comparing the cost-effectiveness of implementable guidelines with other approaches for promoting guideline use. While various bodies of literature describe the infrastructure needed to support collaborative research, there is no definitive model that may be applicable to all contexts. Needs assessment and evaluation of GIRAnet will provide greater understanding of how to develop and sustain such knowledge-exchange networks. Ultimately, by facilitating use of guidelines, this research may lead to improved delivery and outcomes of patient care.

## Competing interests

The authors declare that they have no competing interests.

## Authors' contributions

ARG conceived the study and its design, acquired funding, and will lead coordination of its conduct. MCB and OKB contributed to study design and will participate in data collection and interpretation. All authors read and approved the final manuscript.
